# A comparative study on the effectiveness of pollutants control measures adopted in the steel industry to reduce workplace and environmental exposure: a case study

**DOI:** 10.1038/s41598-024-60817-w

**Published:** 2024-04-30

**Authors:** Daniel Onut Badea, Alina Trifu, Doru Costin Darabont

**Affiliations:** National Research and Development Institute on Occupational Safety - I.N.C.D.P.M. “Alexandru Darabont”, 35A Ghencea Blvd., 061692 Bucharest, Romania

**Keywords:** Electric arc furnace (EAF), Occupational health, Pollutant capture system, Steelmaking process, Environmental sciences, Health occupations

## Abstract

Our understanding of the environmental and occupational health implications of pollutants emitted in steel production is still lacking, despite the considerable amount of research devoted to this topic. Given the significance of steel recycling and the need to reduce greenhouse gas emissions, many steel factories are adopting electric arc furnace (EAF) technology. The use of a technological system designed for the capture of pollutants emitted through EAF steel production is highly ecological because of its utilization of iron scrap and low investment cost. Despite this, the main issue with the EAF is the environmental impact it poses, specifically the release of pollutants into the air, such as dust and organic substances, chlorinated dioxins and furans, dioxin-like polychlorinated biphenyls and brominated dioxins and furans. As a result, workers in this field have a considerable rate of morbidity. The main challenge for EAFs is to optimize the capture of powders produced during the techno-logical process, both from the EAF and the workplace. A state-of-the art solution for managing pollutants in modern steel manufacturing is highlighted in this paper, featuring a method used in Romania that employs the Best Available Techniques (BAT) reference document for iron and steel production to directly collect pollutants from the EAF. The system included a cylindrical fitting, a heat exchanger to cool the gases and a hood to collect contaminants. In comparison to other ventilation options, this equipment boasts lower investment and lower operational costs because of its effective and minimal air flow. Through the use of cutting-edge technology and progressive strategies, we can move closer toward our objective of a workplace free from injuries in the steel industry.

## Introduction

Iron and steel have been vital for the development of human civilizations for thousands of years. They have been used in various fields such as agriculture, construction, machinery and equipment manufacturing, households or medicine. Nine billion metric tons of crude steel were produced on a global scale in 2022^1–4^. The remarkable durability, versatility and recyclability of steel make it an essential resource in today’s world. Owing to the growing need for eco-friendly steel production in light of current environmental conditions, the method of steel production has gradually transitioned from traditional furnaces to EAF ^5^. To achieve sustainable steel production, innovative production technologies and recycling methods for metals and steel must be incorporated ^6^.

Today, steel is produced via the melting of metal in EAF. Despite the benefit of expeditious melting of metals, the main issue with EAFs is the environmental impact they have, specifically the release of pollutants into the air and soil ^7,8^. Steel production in EAF is limited by the considerable number of contaminants (toxic gas and dust) discharged during the melting process. The EAF is equipped with openings designed for electrode passage, which effectively removes contaminants from the EAF. However, this process can also lead to pollution of the workplace and surrounding environment due to the evacuation of melted metal through the top and discharge pan. In addition to the release of pollutants, a considerable amount of energy is discharged through convection and radiation. Electric arc furnaces used for direct smelting of iron-containing materials, mainly from scrap, demand a large amount of electricity and generate air pollutants and solid residues including scrap, filter dust, and slag. The furnace contains a broad range of inorganic compounds (e.g., iron oxide dust and heavy metals) and organic compounds, for example, persistent organic pollutants.

Pollution emitted from EAFs has had a major impact on the environment. According to Chi et al., the most damaging pollutants originating from the EAF are powders created during base material loading, steel melting, refining, alloying and evacuation processes. These powders contain heavy metals such as Cr, Ni, Zn, and Pb ^9^. The composition of the powders may, change in response to certain stipulations of the EAF process, such as the feedstock composition, furnace temperature and production period ^10^.

The gases produced as a result of melting and refinement operations are predominantly composed of CO, SOx and NOx ^11^. During the smelting process, elements such as Zn, Fe, Cr, Mn and Pb, commonly found in iron scrap, can become volatile at temperatures as high as 1600 °C. A significant amount of energy is used in the manufacture of iron and steel. CO_2_, a greenhouse gas, is produced when energy is utilized. Steelmaking processes necessitate sufficient temperature to catalyze chemical reactions and physical treatment, a reductant to reduce iron oxide, and energy and steam to drive steel mills, all of which contribute to CO_2_ emission. A considerable amount of unwanted dust, known as electric arc furnace dust, is produced in the vapor stage during the cooling of an EAF. A dust collection system is employed to systematically gather this industrial byproduct ^12^. For EAF steelmaking, the following techniques or combination of techniques are considered as BAT: a combination of direct off gas extraction (4th or 2nd hole) and hood systems or dog-house and hood systems or total building evacuation. At least 98% of the primary and secondary emissions from the EAF can be effectively collected^13^. In most cases, an air bag filters are utilized to eliminate any dust particles from the exhausted air before it is discharged into the stack.

The different types of pollutants produced by an EAF can be found in Table 1.

**Table 1:** The nature of the pollutants emitted by an electric arc furnace.

Pollutants	Nature of the pollutants
Toxic gases	When oxygen or argon is used for insufflation, carbon monoxide becomes the most prominent gas, making up approximately 90% of the emitted gases. It has been observed that sulfur dioxide, a poisonous gas, can be produced during certain melting processes. In addition to these gases, there are also some that pose a reduced threat to human health, namely, carbon dioxide, oxygen, nitrogen, and various metal oxides in the form of vapors and gases that result from the combustion of auxiliary components used in the EAF along with the melting materials, substances such as oils, paints, natural materials, man-made resins, and remnants of animal or plant matter. There is a significant fluctuation in the percentage of different gases in the melting process. During the melting process, carbon dioxide initially sees a steep increase, then a decline, followed by another rise during loosening, and ultimately a decrease. The upper area of the EAF space is where carbon monoxide is produced, along with the entrainment of solid particles and vapors. The ongoing operation of the EAF produces a constant stream of carbon monoxide
Powders (dust)	It is estimated that the production of one ton of steel produces approximately 15–30 kg of dust ^14^. One of the numerous process residues produced during the steel manufacturing process is electric arc furnace dust (EAFD). EAF dusts are generally characterized by significant levels of Zn, Fe, and Pb, along with varying levels of Cd, Ca, Mg, Cr, Mn, Si, Ni, Cu, F, and Cl. EAFD, is generated as a result of the vaporization of molten iron with nonferrous metals, CO bursting bubbles and the ejection and dragging of particles from the metal bath, slag and other materials in the EAF ^15^. Guézennec et al. found that EAFD is created when particles are vaporized at high temperature regions within the furnace, such as the arc, and when carbon monoxide bubbles burst in the steel bath, resulting in the formation of dust. This dust is then carried in the form of vapor to the gas extraction system^16^. EAF powder can be classified into two groups based on size: particles ranging from 0 to 5 μm and particles ranging from 5 to 50 μm. Oxides of iron, calcium, silicon, manganese, magnesium, zinc, chromium, copper, and aluminum make up the majority of the powders, with an average carbon dioxide content of 40–60%. After conducting a distinct study on iron oxides, it was determined that when oxygen is not introduced, particles smaller than 5 μm make up approximately 60 to 70% of the mixture. However, when oxygen is injected during the process, the percentage of small particles significantly rises to 90%, resulting in the formation of “brown smoke” from Fe_2_O_3_ and “black smoke” from Fe_3_O_4_. Based on this evidence, we can conclude that the addition of oxygen in an EAF result in a higher production of fine smoke, posing a greater challenge in its separation from the gases. Typically, dimensions below 1 μm and in the case of EAF for the elaboration of some steels with alloys that contain silicon, powder particles of only 0.1 μm, represent over 80% of their entire quantity, their retention from gases constituting a difficult problem to solve. The smoke generated during the production of silicon alloys has a gray hue and is rich in SiO_2_ and carbon from the electrodes. The gas within the EAF is registering a significantly high temperature (roughly 1200 °C), resulting in intense impacts and releasing excessive smoke. The smoke produced by manganese alloys has a brown appearance due to the presence of manganese and silicon oxides. In the presence of chromium alloys, the smoke produced is light in hue, typically varying from white to a faint color, and contains a combination of MgO, SiO_2_, iron, and chromium oxides. The nature of waste and other input materials are important factors in defining the chemical composition of the particles carried by the gases and the overall appearance of the smoke. EAF’s raw materials consist mainly of scrap steel and iron, along with scrap rubber. Pre-impurities, such as phosphorus (P), aluminum (Al), manganese (Mn), and silicon (Si), are often found in steel scrap. Recently, there has been an increase in chlorine levels, which can be attributed to a higher number of Cl-containing impurities from various sources, including scrap metal, tires, paints, and polymers, as well as inorganic materials often accompanying waste, such as NaCl and KCl. As a result of the thermal decomposition of impurities through the electric arc's heat, some of the chlorine (Cl) is transferred to EAFD, resulting in the formation of alkaline metal chlorides. This process often results in chlorine being the most prevalent element in EAFD, second only to iron (Fe), zinc (Zn), and oxygen (O)^17,18^. Iron oxides (Fe2O3) and calcium oxide (CaO) are formed through the use of Fe-based materials and lime in the furnace and slag. The presence of ZnO in the dust is determined by the quantity of galvanized steel in the scrap. During the smelting process, Zn is transformed into a gaseous form, and leaves the metal bath, but later becomes oxidized into ZnO when it combines with the waste gas and encounters certain oxidation conditions ^19^. The carbon emissions during steelmaking can be attributed from various sources, such as the carbon present in the charge components like old scrap, pig iron, and graphite electrodes ^20^

The operations involved in the EAF steelmaking process, such as waste loading, smelting and refining, steel and slag tapping, and furnace and ladle lining repairs, all contribute to the generation of emissions. These emissions are composed of iron oxide, calcium oxide, and nitrogen oxides, with variations depending on the specific process and waste and other input materials used. The emissions from waste loading are challenging to measure due to variations in the waste grade, while refining and tapping both release iron oxide as a major particulate compound. The furnace flue gases, which are mainly composed of carbon monoxide, are emitted from the top of the furnace and contain particles. An overview of the air released in the different sections of the steel preparation process can be found in Table 2.

**Table 2:** Air release routes for pollutants emitted by an electric arc furnace.

Potential release	Charging process	Smelting and refining process	Steel and slag tapping process	Furnace and ladle lining repairs process
Carbon dioxide (CO2)	Emit into atmosphere	Emit into atmosphere		
Carbon monoxide (CO)	Emit into atmosphere	Emit into atmosphere		
Sulphur dioxide (SO2)	Emit into atmosphere	Emit into atmosphere		
Nitrogen oxides (NOx)	Emit into atmosphere	Emit into atmosphere	Emit into atmosphere	
Iron oxide (Fe_2_O_3_)			Emit into atmosphere	Emit into atmosphere and to land
Calcium oxide (CaO)		Emit into atmosphere		
Hydrogen chloride (HCl)	Emit into atmosphere			
Hydrogen fluoride (HF)		Emit into atmosphere		
Zinc (Zn)	Emit into atmosphere	Emit into atmosphere		
Slag		To land	To land	Emit into atmosphere and to land
Other metals and their oxides			Emit into atmosphere	
Other inorganic chemicals	Emit into atmosphere	Emit into atmosphere		Emit into atmosphere and to land
Metallic iron				Emit into atmosphere and to land
Inorganic fluorides				Emit into atmosphere and to land

Dealing with steel involves a procedure that carries inherent hazards, thus requiring careful management. The steps necessary to regulate these risks can be quite complicated. As a result, workers in this field have a considerable rate of morbidity. Chronic bronchitis, asthma, and pulmonary tuberculosis followed by upper respiratory tract infections are the most common respiratory issues. The main allergenic components are nickel, chromium, iron, and copper in the form of metal dust. A sampling of the work environment was conducted to ascertain the types of pollutants to which workers are exposed ^21,22^.

## Materials and methods

The operational data of a 10 t EAF were utilized for this study. The main focus of this paper is to conduct a detailed investigation on the collection of hazardous waste, specifically EAF dust, at a steel production plant in Romania. With a design for production (DFP) approach, the research method utilizes research techniques to analyze the design process of pollutant capture technology, taking into consideration the multilevel system perspective of steel production activities. The research was conducted using experimental approaches, spanning from laboratory research to investigations at the factory level. The DFP approach includes assessing the effectiveness of the pollutant capture technology used in steel production. The data for this research was collected during a regular EAF operation, without any intentional or preplanned experiments being conducted. The findings were then compared to the designated emission limits for these pollutants, as indicated in the Ref.^22^.

**Figure 1:**
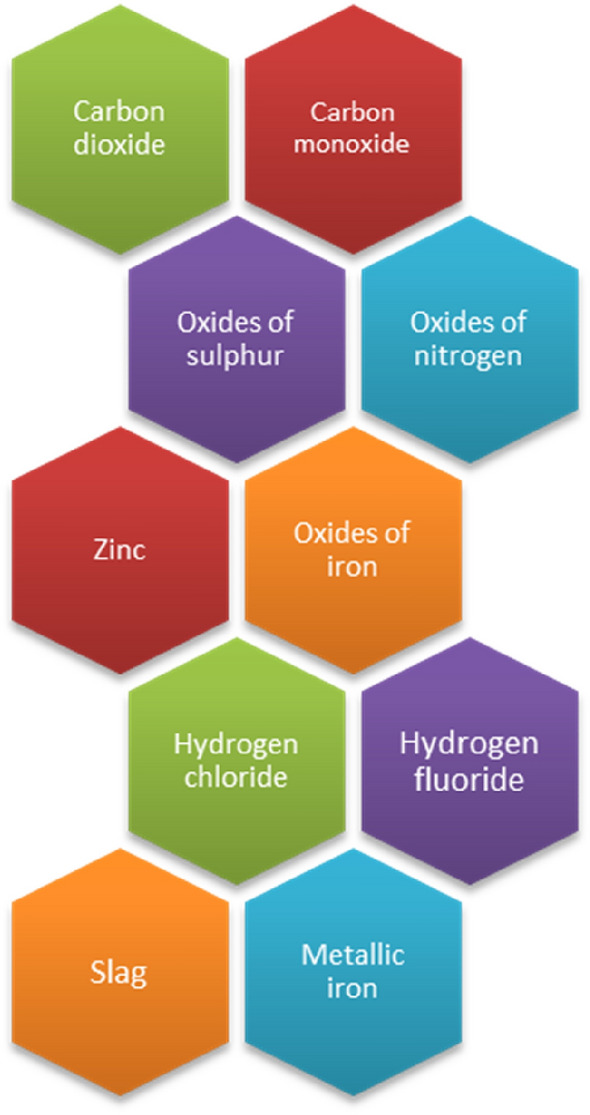
Schematic representation of the pollutants emitted by an electric arc furnace.

The first stage of the research involved identifying and examining the pollutants that were being emitted in the workplace and the surrounding environment. Figure 1 presents a schematic representation of the pollutants emitted by an EAF.

Along with carbon oxide, sulphur dioxide, carbon dioxide, nitrogen oxides, and various metal oxides in vapor form, the EAF used for steel manufacturing also exhausts other gases that are generated from burning the auxiliary products introduced in the EAF, such as oils, paints, and organic materials.

Electric arc furnace emissions include dust particles measuring between 0.1 and 1 μm and dusts entrained by gases and fumes of sizes up to 50 μm ^23^.

The chemical structure of the gases that are entrained fluctuates based on the substances included in the charge. Iron, calcium, silica, manganese, zinc, chrome, copper, aluminum, and similar materials are typically present in dust particles. The volume of gas vented from an EAF is dependent upon the charge material, work system, blast oxygen amount, blast duration, aeration degree, furnace structure, and many other factors. The gases that exit of the EAF reach a temperature of approximately 1300 °C while melting and 1500 °C when oxygen aeration is performed or when carbon oxide emitted from the EAF encounters air at the arch of the furnace, leading to a rise in gas temperature.

The absence or malfunction of operation of the ventilation equipment has revealed, through measurements taken at the EAF, an excessive number of contaminants in the vicinity of the furnace and its housing, carried by the air draught. By means of skylights, gases and dust are discharged into the environment, resulting in severe pollution. The concentrations of pollutants in the work environment during different phases of the process exceeded 3–15 times the accepted concentration values.

Analysis of the granulometric spectrum of the suspension particles revealed that 32–45% were submicronic, 53–63% were between 1 and 3 μm, and less than 1% were between 3 and 5 μm.

The conducted studies revealed the divergence of contaminants arising from technological processes. Over a span of 24 h, a 3 t EAF with a capacity of 600 kg of dust of sizes between 0.1 and 12 μm was exhausted. As a consequence, pollutants emitted into the atmosphere (including dust, metal oxides, nitrogen oxides, carbon dioxide, and sulphur dioxide) have caused pollution which is a major threat to the environment.

After laboratory and in situ studies, a novel system for collecting contaminants with decreased air flow and superior effectiveness was developed. The working method of the technological system is to capture all types of pollutants that come out of the EAF. The following procedures were used to collect contaminants. The contaminants released during the manufacturing process were collected by direct removal from the furnace arch. The addition of a hood above the EAF ensures that all pollutants produced during the feeding and discharging process are efficiently eliminated.

## Results

Five filter materials were tested in the laboratory to determine the most suitable materials for trapping powder released during the steel production process in a 10 t EAF. Table 3 presents the technical manufacturing features of the filter materials.

**Table 3:** Technical manufacturing features of the filter materials.

Sample	Fibrous composition	Specific mass			
		Finite	Fiber	Binder	Wire support
A	100% PES tip L + B woven insert laminator Nonwoven	400	252	–	148
		520	372	–	148
B	100% PES tip L nonwoven insert Nonwoven	400	252	–	148
		520	372	–	148
		600	452	–	148
C	100% PES tip L Nonwoven	500	400	100	–
D	100% PE woven	210	210	–	–
E	Polyester fiber with polyester insert nonwoven	540	540	–	–

The performance of a filter is predominantly determined by the technical specifications of the filter material utilized in the production of the bags that equipped the filter. Proper selection of material for filter bags is essential for ensuring efficient dust removal, reliable filtration, low energy consumption, and manageable investment and operation costs depends on their behavior under real operating conditions. Besides filter media quality mechanical quality (gaps) of filter house is essential for proper environmental performance. Laboratory tests were conducted to assess the effectiveness of filter materials on the powders released from the 10 t EAF during steel elaboration and collected on the machines. The distribution of dust granulometry and the average particle size were assessed. The granulometric distributions of the powders utilized in the measurements are outlined in Table 4.

**Table 4:** The granulometric spectrum of the suspended powders collected from the 10 t furnace area.

The granulometric spectrum of the suspended powders (%)					
Under 1 μm	1–3 μm	3–5 μm	5–10 μm	10–20 μm	Over 20 μm
41.02	55.86	2.02	0.78	0.32	–

Granulometric analysis was conducted with powders in suspension collected under loading conditions 60 min. after the melting phase started and the furnace was emptied. There were identified two categories of particles: fine particles under 1 μm, and coarse particles with sizes ranging from 1 to 20 μm. The majority of the particles analyzed have a size that is less than 10 μm. None of the particles were identified as being over 20 μm. The analysis reported two size categories: fine particles below 1 μm and coarse particles ranging from 1 to 20 μm.

The comparison of the findings for the four dust samples collected from the steel mill reveals the diversity in the composition of EAF dust, influenced by the operational parameters of the EAF melting process and the type of scrap used, in line with existing literature ^24^.

Through laboratory testing, the following parameters were identified: the air permeability of the filter material in its clean state, the air permeability of the filter material when clogged, and the effectiveness of dust containment. A clean state of the material is necessary to determine its air permeability, measured in l/m^2^ s, which reveals the maximum air flow that can pass through the filter at a steady pressure loss (200 Pa). As stated before, the air permeability of the clogged filter material remains unchanged. To confirm this, successive loading and shaking steps were used to fill the material mounted on the test stand with powders. A calculation was performed to determine the retention efficiency of the powders when filtered through the material:

$$ {\upeta } = \frac{{{\text{C}}_{{\text{i}}} - {\text{C}}_{{\text{e}}} }}{{{\text{C}}_{{\text{i}}} }} \times 100\,\left( \% \right), $$

where c_i_—dust concentration at the filter entrance (g/m^3^), c_e_—dust concentration at the filter exit (g/m^3^).

A total of five filter materials were tested. The central representation of the experimental findings can be found in Table 5.

**Table 5:** Results obtained during the testing of filter materials.

Filter materials sample	Mass per unit area (according STI) (g/m^2^)	Sample number	Mass per unit area (g/m^2^)	Permeability to air (l/m^2^ s)		Filtration efficiency η (%)	
				Clean p_1_	Residual loading p_2_	On sample	Average
A	400	I	397	405	304	84.7	85.6
		II	410	390	293	85.5	
	520	I	505	360	280	86.9	88.7
		II	530	340	272	90.5	
B	400	I	405	505	399	87.3	87.4
		II	420	410	324	87.5	
	520	I	520	360	285	88.1	88.9
		II	540	350	277	89.7	
	600	I	605	320	253	88.7	89.2
		II	620	300	237	89.7	
C	500	I	495	1150	1053	97.3	87.6
		II	540	1100	1015	98.1	
D	210	I	208	920	912	93.6	94.1
		II	217	935	925	94.6	
E	540	I	540	1050	1027	98.7	99.1
		II	555	1055	1021	99.5	

Proper filtration of dust-laden gases from 10 t EAF requires initial cooling. The purpose of cooling gases with powders is to safeguard the filter from potential fires caused by the powders. To prevent the burn of the filter bags, the gases carrying powders, which have a temperature of approximately 1100 °C when entering the furnace’s suction mouth, must be cooled to a maximum temperature of 120 °C. The installation’s design guarantees a two-stage cooling process for gases that contain powders. In the first stage the gases loaded with powders were cooled to an initial temperature of 1100 °C to a final temperature of 350 °C by mixing with the atmospheric air drawn from the hall. Using a heat exchanger, the second stage lowers the temperature of the gas-powder mixture from 350 °C to a filter inlet temperature of 120 °C. The first stage of cooling involves mixing atmospheric air, which has an average temperature of approximately 30 °C, with the hot gases captured from the EAF before they are introduced into the heat exchanger. The EAF is equipped with a hood situated in the hall for efficient intake of atmospheric air. Removing air from the hall with the hood has positive effects as it helps capture the gases emitted by the EAF during loading and when the vault is removed. The hall utilizes a hood to facilitate the intake of atmospheric air for the EAF. Pulling the air out of the hall using the hood has advantages because it effectively captures the fumes produced by the EAF during loading and when the vault is removed. The inside of the bundle of pipes is filled with hot gases at a scorching temperature of 350 °C, while the outside is cooled by circulating air that absorbs heat. Once the hot gases travel through the bundle of pipes in a double circuit, they enter and exit the cooler at the top. In the cooler, atmospheric air is directed toward the lower side and travels through three circuits, cooling the pipes with fins, before exiting from the upper side. The heat balance equation is used to calculate the flow rate of atmospheric air required to cool gases carrying powders in the hall^12^:

$$ {\text{Q}}_{{\text{g}}} + {\text{ Q}}_{{{\text{co}}}} + {\text{ Q}}_{{\text{a}}} = \, \left( {{\text{V}}_{{\text{g}}} + {\text{ V}}_{{\text{a}}} } \right) \cdot {\text{c}}_{{{\text{pam}}}} \cdot {\text{t}}_{{{\text{am}}}} \left( {{\text{kcal}}/{\text{h}}} \right), $$

where ${\text{Q}}_{{\text{g}}} = {\text{ V}}_{{\text{g}}} \cdot {\text{c}}_{{{\text{pg}}}} \cdot {\text{t}}_{{\text{g}}} \left( {{\text{ kcal}}/{\text{h}}} \right)$—the heat contained in the gases drawn from the furnace, ${\text{V}}_{{\text{g}}} = { 39}0 \cdot {\text{C}}^{{0.{73}}} \left( {{\text{ Nm}}^{{3}} /{\text{h}}} \right)$—the flow of gases released during the elaboration of steel batches, ${\text{C }} = { 1}0 \cdot \left( {\text{t}} \right)$—the maximum capacity of the furnace, ${\text{V}}_{{\text{g}}} = { 39}0 \times {1}0^{{0.{73}}} = { 2}0{94 }\left( {{\text{Nm}}^{{3}} /{\text{h}}} \right),$ ${\text{c}}_{{{\text{pg}}}} = \, 0.{3695 }\left( {{\text{ kcal}}/{\text{Nm}}^{{3}} \;^\circ {\text{C}}} \right)$—the specific heat of gases, ${\text{t}}_{{\text{g}}} = { 11}00 \, \left( {^\circ {\text{C}}} \right)$—the temperature of the gases at the exit from the furnace.

By substituting the values in relation, we obtain:

$$ {\text{Q}}_{{\text{g}}} = { 2}0{94} \times 0.{3695} \times {11}00, $$

$$ {\text{Q}}_{{\text{g}}} = { 851}.{279 }\left( {{\text{kcal}}/{\text{h}}} \right), $$

$$ {\text{Q}}_{{{\text{co}}}} = \, 0.0{9} \times {\text{V}}_{{\text{g}}} {\text{H}}_{{\text{i}}} \times \left( {{\text{kcal}}/{\text{h}}} \right), $$

where ${\text{Q}}_{{{\text{co}}}} \left( {{\text{kcal}}/{\text{h}}} \right)$—the heat released by burning CO at the exit from the furnace, ${\text{H}}_{{\text{i}}} = {3}000 \, \left( {{\text{kcal}}/{\text{Nm}}^{{3}} } \right)$ calorific value of CO.

By substituting the values in relation, we obtain:

$$ {\text{Q}}_{{{\text{co}}}} = \, 0.0{9} \times {2}0{94} \times {3}000, $$

$$ {\text{Q}}_{{{\text{co}}}} = { 565}.{38}0 \, \left( {{\text{kcal}}/{\text{h}}} \right), $$

$$ {\text{Q}}_{{\text{a}}} = {\text{ V}}_{{\text{a}}} \times {\text{c}}_{{{\text{pa}}}} \times {\text{t}}_{{\text{a}}} \left( {{\text{kcal}}/{\text{h}}} \right), $$

where ${\text{Q}}_{{\text{a}}} \left( {{\text{kcal}}/{\text{h}}} \right)$—the heat contained in the air drawn from the hall, ${\text{V}}_{{\text{a}}} \left( {{\text{Nm}}^{{3}} /{\text{h}}} \right)$—the flow of air drawn from the hall, ${\text{c}}_{{{\text{pa}}}} = \, 0.{31 }\left( {{\text{kcal}}/{\text{ Nm}}^{{3}} \;^\circ {\text{C}}} \right)$—specific heat of air, ${\text{t}}_{{\text{a}}} = { 3}0 \, \left( {^\circ {\text{C}}} \right)$—the air temperature in the hall.

By substituting the values in relation, we obtain:

$$ {\text{Q}}_{{\text{a}}} = {\text{ V}}_{{\text{a}}} \times 0.{31} \times {3}0, $$

$$ {\text{Q}}_{{\text{a}}} = { 9}.{\text{3 V}}_{{\text{a}}} \left( {{\text{kcal}}/{\text{h}}} \right). $$

For the temperature of the mixture (gases + air) of ${\text{t}}_{{{\text{am}}}} = { 35}0\;^\circ {\text{C}}$ and ${\text{c}}_{{{\text{pam}}}} = \, 0.{3262}\left( {{\text{kcal}}/{\text{ Nm}}^{{3}} \;^\circ {\text{C}}} \right)$, thermal balance equation becomes:

$$ {851}.{279 } + { 565}.{38}0 \, + { 9}.{\text{3 V}}_{{\text{a}}} = \, \left( {{2}0{94 } + {\text{ V}}_{{\text{a}}} } \right) \times 0.{3262} \times {35}0, $$

where $${\text{V}}_{{\text{a}}} = { 11}.{229 }\left( {{\text{Nm}}^{{3}} /{\text{h}}} \right)$$.

The relation provided below allows for the calculation of the total flow rate of the gas and air mixture:

$$ {\text{V}}_{{\text{T}}} = {\text{ V}}_{{\text{g}}} + {\text{ V}}_{{\text{a}}} \left( {{\text{Nm}}^{{3}} /{\text{h}}} \right), $$

$$ {\text{V}}_{{\text{T}}} = { 2}0{94 } + { 11}.{229 } = { 13}.{323 }\left( {{\text{Nm}}^{{3}} /{\text{h}}} \right). $$

Cooling of gas + air flow ${\text{V}}_{{\text{T}}} \, = \,{13}.{323 }\left( {{\text{ Nm}}^{{3}} /{\text{h}}} \right)$ and a temperature of 350 °C are achieved in a pipe heat exchanger to a temperature of 120 °C.

The calculation of the required air flow to cool the gases in the cooler of a tubular heat exchanger is as follows:

$$ {\text{V}}_{{{\text{aer}}}} = { 36}00 \times {\text{w}}_{{\text{e}}} \times {\text{S}}_{{{\text{aer}}}} \left( {{\text{m}}^{{3}} /{\text{h}}} \right), $$

where ${\text{w}}_{{\text{e}}} = { 1}0 \, \left( {{\text{m}}/{\text{s}}} \right)$—air velocity outside the finned tubes, ${\text{S}}_{{{\text{aer}}}} {-}\left( {{\text{m}}^{{2}} } \right)$—the free surface area for air to pass through the outer finned pipes of the selected heat exchanger is $0.{\text{5724 m}}^{{2}}$.

$$ {\text{V}}_{{{\text{aer}}}} = { 36}00 \cdot {1}0 \cdot 0.{5724 } = { 2}0,{6}06{\text{ m}}^{{3}} /{\text{h}}. $$

The heat exchanger will be equipped with a fan that meets the following technical requirements:

$${\text{debit}}\, = \,{21}.000{\text{ m}}^{{3}} /{\text{h,}}$$

$${\text{power}}\, = \,{18}.{\text{5 kW}}.$$

An installation flow rate of ${4}0,000{\text{ m}}^{{3}} /{\text{h}}$ is achieved due to the connection between the heat exchanger and the pipeline that carries the extracted gases from the hood.

**Figure 2:**
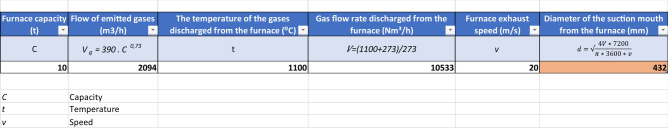
Software tools for calculating the EAF outlet size.

Using Microsoft Excel, the authors designed a software tool to calculate the furnace outlet size for a 10 t furnace, based on the mathematical equations outlined above. The spreadsheet’s caption is displayed in Fig. 2.

The examination and contrast of test outcomes revealed that directly capturing gases from the furnace is the most efficient approach. The system for collecting contaminants directly from the EAF involves a cylindrical fitting and a pipe. The span of the furnace arch is a cylindrical fit. Its diameter is determined based on the EAF’s capacity.

**Figure 3:**
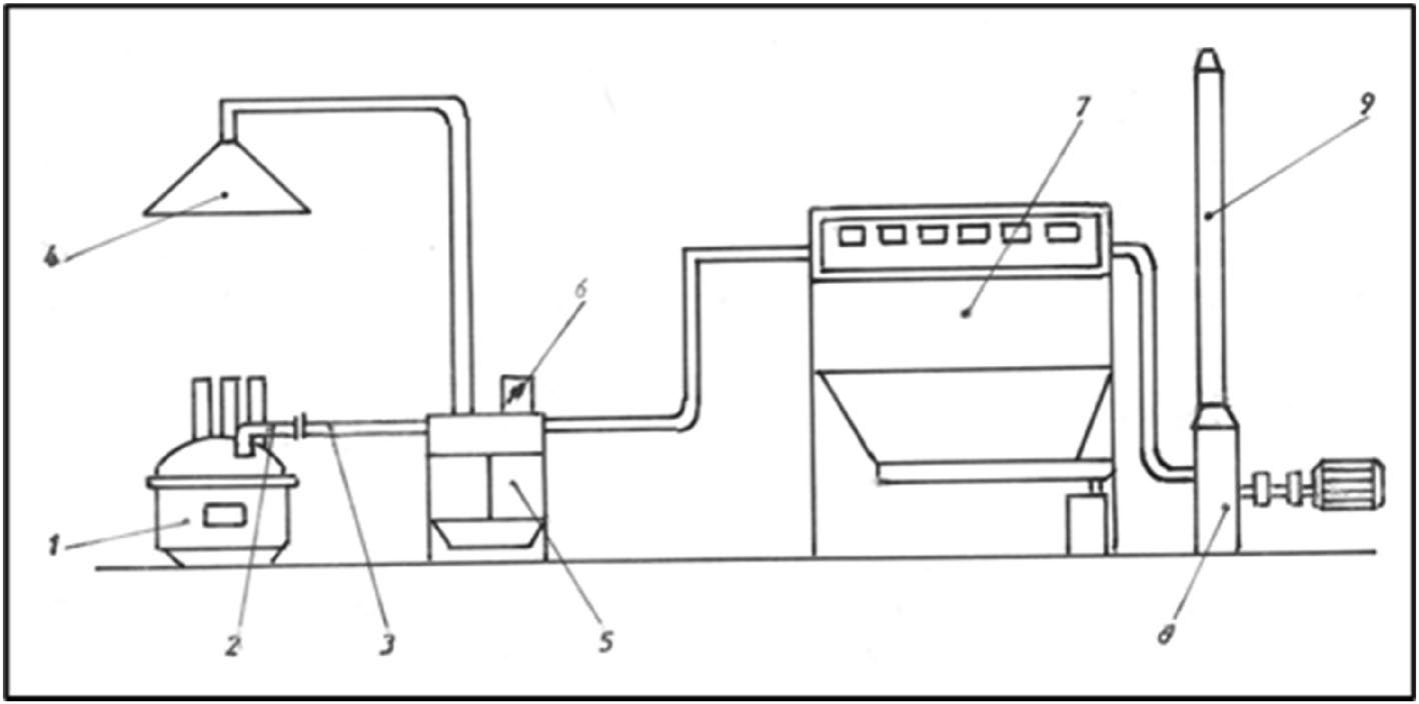
Prototype of the pollutant collector system for a 10 t electric arc furnace.

A type of off-gas collection system for EAF with 4th hole extraction systems was design, with four basic elements: the hood, the duct system, the air cleaning device, and the fan. Figure 3 illustrates the design of the prototype application of a pollutant collector from a 10 t EAF. The design of this system was based on the following collection configuration:

- a fitting (2) with a diameter of 450 mm that crosses the EAF (1) to remove the pollutants;
- a pipe (3) with an adjustable end placed at the end of the fitting (2). This allows for the intake of false air from the hall, and by varying the distance between the fitting (2) and pipe (3), the gas pressure in the EAF can be regulated to draw in false air for the purpose of cooling the gases and burning CO. By adjusting the EAF pressure, the minimum flow needed to capture the residual gases is ensured. By adjusting the distance between the fitting (2) and the pipe (3), the amount of gas absorbed from the EAF can be controlled (increased or decreased), causing the pipe to remain fixed when a small quantity of smoke is visible in the area of the electrodes coming out of the EAF and is drawn in by the hood (4).
- a hood (4) that sucks the gases leaked from the EAF while discharging and feeding the EAF. Furthermore, it ensures the cooling of gases to 350 °C.
- a heating exchanger for gas cooling (5), where the temperature is reduced to 120 °C, a temperature that permits filtering without damaging the filtering material the sacks are made of. It has the potential to function as a heat recovery system;
- a thermocouple for measuring the entering temperature in the filter and a flap stump with an automatic adjustable valve (6) for controlling the temperature of the gas entering the filter;
- sack filter (7), having a 525 m^2^ surface for filtration, to retain the solid particles from the gas with pneumatic shaking by an inverted blow of automatic command. The sacks are made of synthetic material and polyester fibers, and resist at a maximum temperature of resisting at the temperature of 140 °C;
- centrifugal fan type (8) with a flow rate of 40,000 m^3^ and 132 Kw electric motor;
- exhaust chimney (9).

Due to space limitations, a doghouse installation was not feasible in this case study.

The EAF is equipped with an automated ventilation system that effectively captures and contains any pollutants emitted, due to its adjustable air flow controls.

Through a set of laboratory tests, the pollutant capture system’s performance was validated by measuring the concentration of contaminants. The system’s effectiveness was assessed before and after installation. The effects of the pollutant capture system on levels of contaminants in the work environment, emissions, and the effectiveness of filter retention were assessed and the findings are summarized in Table 6.

**Table 6:** Analysis of the pollutant capture system's efficacy pre and post installation.

Sample no.	Contaminant concentration (mg/Nm^3^)						Filtering efficiency (%)
	Work environment				Ventilation pipe		
	Before deploying the pollution capture system		After deploying the pollution capture system		Before the filter	After the filter	
	CO	Total dusts	CO	Total dusts	Total dusts	Total dusts	
1	140	18	4	0.25	250	4.5	98.2
2	160	20	5	0.27	241	4.8	98.0
3	170	21	6	0.26	261	4.9	98.1
4	140	19	5	0.25	255	4.8	98.1
5	150	20	5	0.24	245	4.7	98.0

## Discussion

The particle emission factors in Tables 4, 5, 6 were derived from laboratory experiments conducted by the authors. To calculate and summarize the particle size distributions for emissions from the particular case study processes in the steel industry, the best available data was consulted as references. The selection of equipment from Table 5 was guided by the size distributions of the suspended powders.

To guarantee a pollutant capture system’s efficient, cost-effective, and safe operation, high temperature dust filtration is essential ^25^. Pulse jet bag filters utilize a several bags to capture dust and other airborne particles, preventing their release into the environment. Pulse jet bag filters are highly efficient in eliminating small particles, such as dust, powder, smoke, and other airborne particles, from industrial exhaust gases emitted during steel manufacturing process. According to Table 5, the bag filters that were tested demonstrated their effectiveness in removing pollutants. In most cases, pulse jet bag filters have the ability to eliminate up to 99% of the particulate matter from particulate laden exhaust gas streams ^26^. When compared to other dust collection methods, pulse jet filtration systems offer numerous advantages such as safeguarding employee well-being and enhancing operational productivity by maintaining a cleaner air environment. The primary benefit lies in the pulse jet’s capacity to perform online cleaning. With an average duration of 100 to 200 μm, the compressed air pulse allows for continuous 24/7 operations without interrupting dust capture and without any need for bags filter cleaning downtime. Despite their simple installation process, they require diligent and ongoing maintenance to ensure optimal performance. Regular cleaning of the bags system can extend the lifespan of the pollutant capture system. Numerous studies demonstrating the widespread application of bags filters in various industries, including steel and iron, owing to their high filtration efficiency provided evidence in favor of this perspective ^27,28^. Dembinski et al. revealed comparable findings, indicating that the resistance to air flow through the filter material is heightened with longer filtration time^29^. Out of all the filter materials tested in current study, sample E had the highest dust removal efficiency at 99.1%.

The data reveals that the performance of bags filters is impacted not just by their primary characteristics, but also by the specific conditions in which they are used, including high levels of dust and small particle sizes. Reduced filtration resistance could result from these factors, but this can be counteracted by increasing the frequency of cleaning pulses.

The granulometry analysis of EAFD, as determined by the laser granulometer, aligns with previous research ^30,31^. With 98.90% of the particles falling between 0 and 5 μm, Table 4 shows a heterogeneous distribution of particle sizes. This irregular distribution is in accordance with findings from Machado et al. and Mantovani et al. ^32,33^. According to Peng et al. ^34^, the presence of spherical agglomerates composed of fine particles or fine particles covering larger particles is likely due to the agglomeration state of the particles. This has been confirmed by several authors, including Rocabois who observed spherical shapes made of metal oxides of spinel type XFe2O4 (X = Fe, Zn or Mn) ^35^.

Since each plant presents its own distinct circumstances, is not practical to generalize conclusions based on specific situations. According to the research, the proposed method of capturing pollutants for primary and secondary collection of pollutants includes using a combination of a direct off gas extraction (4th hole) and hood systems. In the present case study, space limitations prevented the possibility of a doghouse or total enclosure being installed over the EAF. The removal of evacuated gases from the EAF involves collecting a mixed solution of gaseous phase collecting through the fourth orifice of the arch, equipped with a cooled linkage, and a hood positioned above the EAF for capturing secondary emissions. The gases are cooled using surrounding temperature air and an air cooler, and then filtered through a bags filter. The required draft is provided by a room exhauster, both for the fourth orifice and the hood, while residual gases are drawn up by a skylight-level hood and directed to a bags filter for dust collection before being emitting through the stack. A focus was conducted on the compatibility between the technologies through an assessment of the gas flow rates. An efficiency of more than 98% in dust collection is achieved with this system. A comparison of dust pollutant concentrations before and after the capture system's installation is illustrated in Table 6. The examination of the measured pollutant levels indicated that they fell within the limits set by current regulations. The pollutant capture system efficiency rate meets the standards set by the BAT reference document for iron and steel production. The emission level associated with BAT for dust is < 5 mg/Nm^3^ on a daily average basis. Along with overall performance, the model also supplies flow rates and temperatures for different streams at specific intervals.

According to this study, the findings align with previous research on pollutant capture methods in steel production. This has led to the introduction of various pollutant capture systems to the steel industry. Based on available data, the vast majority of EAF operate according a standard procedure of mixing the furnace dust with air, heating the combustible materials, and cooling it to a temperature under 200 °C ^36^. The conclusions and characteristics align with the findings published by Voicu, which indicate that 41,000 m^3^/h of air flow is used in a 5 t EAF for gas evacuation ^12^.

A study conducted by Plickert yielded comparable findings, showing that implementing a combination of direct fume extraction and a hood system led to significant improvements in the working conditions and a decrease in diffuse dust emissions through roof fans from Ref.^37^. Rentz ^38^, Lindfors et al. ^39^, Dornseiffer et al. ^40^, and Eurofer ^41,42^ all reported similar findings.

By utilizing a heat exchanger to cool the gases and extracting pollutants directly from the EAF, the system presents numerous benefits. The key advantage of the pollutant collector system is to collect pollutants produced by the EAF, while use a minim air flow from EAF. The minimum flow of air drawn from the EAF is the result of the dimensioning of the fan with a reduce energy consumption and a filter with a reduced filtering surface. Despite requiring durable equipment to handle challenging operating conditions, high temperatures, and considerable dust levels, the system provides the benefit of a simple configuration for air handling and piping.

There are two categories of study limitations: conceptual constraints and pollution control system limitations. The first constraint implies that the system’s thermodynamics and economics have not been mathematically modeled. Market diversification and technological innovation contribute to the varied and distinctive nature of steel plants, making it challenging to predict and prevent inaccurate calculations. The second limitation refers to the system’s composition of multiple pieces of equipment, which consists of various pieces that necessitate a preliminary investigation. As a result, the system’s components were seen as enigmatic entities in order to highlight potential challenges in their functionality and efficiency.

## Conclusion

The main goal of this study is to offer a brief description of a commonly utilized technology in the Romanian steel industry and to provide an overview of one of the latest generation technologies of a frequently encountered industry worldwide. Through the case study methodology, the emphasis was placed on the endeavors of Romanian companies in this industry to become environmentally conscious and safeguard the occupational health of their employees. Sustainable environmental development requires a thorough examination of resource efficiency. The use of resources in an efficient manner is associated with higher levels of innovation, improved productivity at a lower expense, and decreased environmental harm. This approach also presents multiple possibilities for reducing emissions and fostering sustainable lifestyles. Thus, the proper management of resources is contingent upon a number of factors, such as the examination of sustainability measures and the allocation of funds for sustainable practices.

Through the use of cutting-edge technology and progressive strategies, we can move closer towards our objective of a workplace free from injuries in the steel industry.

## Data Availability

All data generated or analysed during this study are included in this published article.
